# Melanopsin Contributions to the Representation of Images in the Early Visual System

**DOI:** 10.1016/j.cub.2017.04.046

**Published:** 2017-06-05

**Authors:** Annette E. Allen, Riccardo Storchi, Franck P. Martial, Robert A. Bedford, Robert J. Lucas

**Affiliations:** 1 Division of Neuroscience and Experimental Psychology, School of Biology, Faculty of Biology, Medicine and Health, University of Manchester, Manchester M13 9PL, UK

**Keywords:** melanopsin, intrinsically photosensitive retinal ganglion cells, dorsal lateral geniculate nucleus, spatial vision, mouse vision, receptor silent substitution, metameric light stimuli, receptive fields

## Abstract

Melanopsin photoreception enhances retinal responses to variations in ambient light (irradiance) and drives non-image-forming visual reflexes such as circadian entrainment [1, 2, 3, 4, 5, 6]. Melanopsin signals also reach brain regions responsible for form vision [7, 8, 9], but melanopsin’s contribution, if any, to encoding visual images remains unclear. We addressed this deficit using principles of receptor silent substitution to present images in which visibility for melanopsin versus rods+cones was independently modulated, and we recorded evoked responses in the mouse dorsal lateral geniculate nucleus (dLGN; thalamic relay for cortical vision). Approximately 20% of dLGN units responded to patterns visible only to melanopsin, revealing that melanopsin signals alone can convey spatial information. Spatial receptive fields (RFs) mapped using melanopsin-isolating stimuli had ON centers with diameters ∼13°. Melanopsin and rod+cone responses differed in the temporal domain, and responses to slow changes in radiance (<0.9 Hz) and stationary images were deficient when stimuli were rendered invisible for melanopsin. We employed these data to devise and test a mathematical model of melanopsin’s involvement in form vision and applied it, along with further experimental recordings, to explore melanopsin signals under simulated active view of natural scenes. Our findings reveal that melanopsin enhances the thalamic representation of scenes containing local correlations in radiance, compensating for the high temporal frequency bias of cone vision and the negative correlation between magnitude and frequency for changes in direction of view. Together, these data reveal a distinct melanopsin contribution to encoding visual images, predicting that, under natural view, melanopsin augments the early visual system’s ability to encode patterns over moderate spatial scales.

## Highlights

• A five-primary display is used to define melanopsin’s contribution to form vision
• Melanopsin extends the spatiotemporal range of the mouse early visual system
• The representation of spatial patterns is deficient when melanopsin is not engaged
• A linear model predicting melanopsin’s contribution to pattern vision is defined

## Teaser

Allen et al. probe the origins of form vision and show that the inner retinal photoreceptor, melanopsin, extends its spatiotemporal range. Melanopsin augments the representation of patterns whose spatial scale is larger than that of ongoing changes in direction of view.

## Results

### Melanopsin-Derived Spatial Receptive Fields

Melanopsin-expressing intrinsically photosensitive retinal ganglion cells (ipRGCs) project to the dorsal lateral geniculate nucleus (dLGN) in mice and primates [7, 8, 9], allowing access to the primary visual pathway. The significance of this arrangement for form vision is not clear. Melanopsin’s accepted function is to encode irradiance, and it has recently been shown that melanopsin adjusts activity in the dLGN according to background (ambient) light [10, 11]. Is that the extent of its contribution to thalamocortical vision? Or alternatively, do melanopsin signals have sufficient spatiotemporal resolution also to encode spatial patterns and, if so, what contribution do they make to form vision?

**Figure 1:**
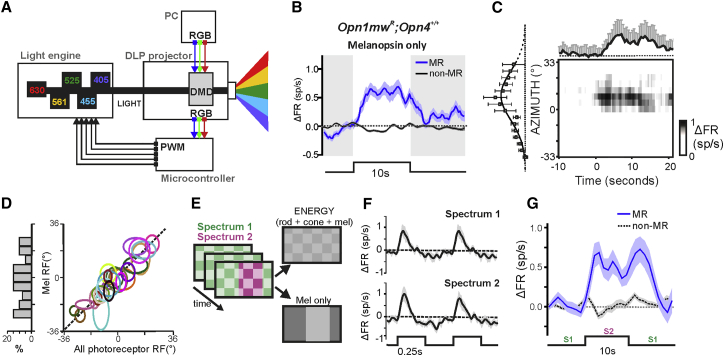
Selectively Activating Melanopsin in Space and Time (A) Spectrally controlled stimuli were generated with a DMD projector in which the intrinsic light source was replaced with a five-primary light engine (LEDs with peak emissions: 405 nm, 455 nm, 525 nm, 630 nm, and a 561 nm laser). To control the light engine, the signal normally sent internally to the projector LEDs is rerouted toward the Chipkit Uno 32 microcontroller. Each color plane of an image (red, green, or blue) is separated in time and synchronized with the PWM control of five LEDs, allowing any combination of the five primaries to be separated in space. (B) Blue line and shading shows mean ± SEM baseline subtracted firing rate (spikes/s) over time of 166 units (out of 668 light response units recorded in dLGN of 25 mice) showing a significant change in firing when presented with a large (72° × 57°) “melanopsin-only” stimulus (10-s presentation of spectrum 2 interleaved with 60-s of spectrum 1) i.e., defined as “MR.” Black line and shading shows mean ± SEM % change in firing rate of 502 units showing no significant change, termed “non-MR.” Timing of stimulus shown below as step and as an interruption of shading on the main plot, dotted line shows baseline activity. (C) Change in firing rate of a representative MR unit as a function of location on the azimuth (at 4.5° resolution) of a 13° “melanopsin-only” bar presented for 10 s. Main plot shows change in firing over time as a heatmap (scale to right) with mean ± SEM firing at the bar position evoking maximal response shown above. The mean ± SEM change in firing rate (at the time of maximum response for optimal bar) as a function of bar location shown to left, Gaussian curve fit (R^2^ = 0.86). (D) Left: histogram of RF centers mapped on azimuth with “melanopsin-only” stimulus. Right: ellipses describing location of RFs on azimuth mapped with “all-photoreceptor” (x axis) and “melanopsin-only” (y axis) stimuli (extent of RF under each condition defined as location on azimuth at half SD on either side of Gaussian fit) for 26 MR units. (E) A cartoon of stimulus projected to the mouse eye: this stimulus consisted of an inverting checkerboard in which high- and low-radiance squares (7.5° squares inverting at 2 Hz, presenting a 3-fold change in radiance) were high- and low-energy versions of spectrum 1. While maintaining presentation of an inverting checkerboard, a change in the spectrum (from spectrum 1 to spectrum 2) was introduced to a large area of the screen. This stimulus therefore presented two concurrent visual stimuli; a high-frequency inverting checkerboard that was spectrally neutral (i.e., visible to all photoreceptors; top right) and a low-frequency spectral change that was only visible to melanopsin (lower right). (F) Mean ± SEM responses (baseline subtracted double plot) of MR-units to high-frequency checkerboard inversions rendered in either spectrum 1 (top) or spectrum 2 (bottom). Firing rates are aligned so that checkerboard inversion evoking maximum response is at time 0. Responses were statistically indistinguishable (Paired t test of response amplitude; p = 0.33). (G) Mean ± SEM firing rate of MR-units (n = 21; black solid line) and non-MR units (n = 81; black dotted line) in response to a transition from spectrum 1 to spectrum 2 (“melanopsin-only”), with superimposed concurrent low-contrast checkerboard inversions. See also Figures S1 and S2.

Addressing these questions with melanopsin knockout or rod+cone-deficient preparations is unsatisfactory. Melanopsin knockout mice have deficits in spatial contrast sensitivity [12], but none of the published physiology indicates that melanopsin has the high contrast sensitivity required for this effect to originate with a direct contribution to encoding patterns, and the effect could plausibly be secondary to more general deficits in visual development and/or function in this genotype [10, 11, 13, 14, 15, 16, 17]. Conversely, while human patients and animal models of advanced rod+cone degeneration have at best rudimentary form vision [18, 19, 20], as ipRGCs are recipients of rod/cone signals their activity would be fundamentally altered in such preparations. A definitive assessment of melanopsin’s contribution to form vision must therefore come from studies of the intact visual system. Recently, we and others have adopted the technique of receptor silent substitution [21, 22] to study melanopsin, using carefully calibrated changes in spectral composition to generate stimuli differentially visible to melanopsin versus rods and cones [13, 20, 23, 24, 25, 26]. This approach achieves the objective of studying melanopsin in animals with intact vision but so far has been restricted to application of spatially diffuse stimuli. In order to extend that analysis to the impact of spatial patterns, we adapted this approach to an apparatus capable of presenting images. In brief, we replaced the light engine of a digital mirror device projector with five independently controllable, spectrally distinct light sources (Figure 1A).

As our first question was whether melanopsin was able to encode patterns, we used the light engine to produce a pair of spectra of equivalent effective intensity for rod and cone opsins but differing for melanopsin (∼52% Michelson contrast). Synchronizing the appearance of these two spectra with the digital mirror device enabled the projection of spatial patterns with much greater contrast for melanopsin versus rods and cones (Figures 1A, 1B, 1F, and S1A–S1C). We confirmed that these spectra were functionally indistinguishable for rods and cones first by showing that they did not elicit responses from melanopsin knockout mice (Figures S1D and S1E). We further validated them by presenting transitions between them at 4 Hz (a frequency to which rods and cones are very sensitive; see the STAR Methods) at the start of each recording session. In all cases, these spectra (or minor adjustments from them) failed to elicit a measurable response at this frequency (Figures S1F–S1K). Insofar as any rod or cone response to these stimuli thus fell below our detection limit, we define them as functionally rod and cone silent (hereinafter termed “melanopsin-only”).

We employed this system to present full screen (occupying 72° × 57° of visual space) “melanopsin-only” steps (10-s duration; 50-s inter-stimulus interval) to anesthetized mice and recorded responses in the contralateral dLGN using a multisite extracellular recording probe. Following spike-sorting, we found that 166/668 light-sensitive single units (from 25 mice) responded to this stimulus with a significant increase in firing (mean ± SEM 1.2 ± 0.08 spikes/s; Figure 1B), equivalent to ∼30% increase from baseline activity (Figure S1C). In agreement with the prediction that these responses originated from melanopsin, they were absent from melanopsin knockout mice (Figures S1D and S1E) and had the poor temporal resolution previously described for melanopsin (Figure 1B).

To determine whether melanopsin can convey spatial information, we next presented a simple “melanopsin-only” pattern (vertical bars; 10-s duration). All units defined as melanopsin-responsive (MR) using the full screen stimulus responded to these bars at some, but not all, locations in visual space. We therefore used these data to describe melanopsin RFs (Figure 1C). In all cases, receptive fields (RFs) comprised an excitatory center without evidence of an inhibitory surround. Accordingly, responses to “melanopsin-only” bars covering a unit’s RF center were similar to those produced by full screen melanopsin stimuli (mean ± SEM 0.92 ± 0.13 and 1.2 ± 0.08 spikes/s, respectively; two-tailed t test; p = 0.163). RFs could be fit with a Gaussian function (mean R^2^ = 0.72) whose width ranged from 4.9° to 21.3° (mean = 13.1°; median = 13.5°) and were distributed across the scene (Figures 1D and S2A–S2C). dLGN responses to “melanopsin-only” bars also had characteristic poor temporal resolution of melanopsin-driven activity (mean ± SEM time to half max firing rate: 5.91 ± 0.73; range: 1–14 s). At the single unit level, RFs mapped using “melanopsin-only” stimuli were equivalent to those mapped using a conventional mapping protocol eliciting responses from rods and cones, confirming that inner and outer retinal photoreceptors convey coherent spatial information to the dLGN (Figures 1D and S2A–S2C). RFs had similar size in MR and non-MR units (Figure S2G), and in both cases, we were unable to map robust inhibitory surrounds (as previously reported in mice [27, 28]).

Responses to “melanopsin-only” bars confirm that melanopsin alone can convey spatial information. A subsequent question is whether it could perform a similar function in more natural conditions in which spatial information is also available for rods and cones. To address this, we applied a 10 s “melanopsin-only” bar (∼33° width) stimulus superimposed on a checkerboard visible to all photoreceptors (square width = 7.5°) inverting at higher frequency (2 Hz; Figure 1E). As expected, the checkerboard stimulus modulated firing in numerous dLGN units (13/21 MR and 61/81 non-MR units; Figure 1F). MR units alone also responded to the “melanopsin-only” bar (Figure 1G). The magnitude of this “melanopsin-only” response was unaffected by the inverting checker (paired two-tailed t test for change in firing rate over 10 s presentation versus “melanopsin-only” bar alone: p = 0.64; mean ± SEM 0.51 ± 0.10 and 0.59 ± 0.14 spikes/s, respectively), indicating the melanopsin-driven response was substantially isolated from that evoked by the higher spatiotemporal frequency stimulus.

### Melanopsin Augments Responses to Static Images

**Figure 2:**
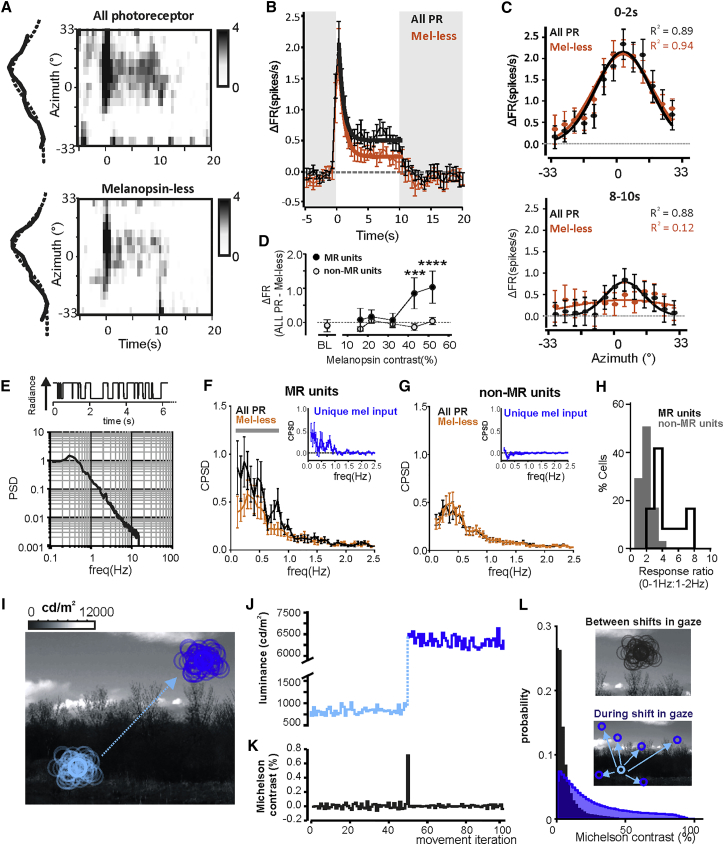
Melanopsin Sustains Responses to Static Images (A) Change in firing rate of a representative MR unit as a function of location on the azimuth (at 4.5° resolution) of 13° “all-photoreceptor” (upper panel) and “melanopsin-less” (lower panel) bars (10 s every 60 s) presented for 10 s. Main plot shows change in firing over time as a heatmap (scale to right) with mean normalized change in firing at the time of peak response as a function of bar location fitted with Gaussian curve (dashed line) to left. (B) Mean ± SEM change in firing rate for “all-photoreceptor” (black) and “melanopsin-less” (orange) bars (10 s starting at time 0) presented at RF center of MR units (n = 40). Solid lines show fit for data from 0–10 s with exponential decay curves. Separate curves were required for the two conditions (F test comparison; p < 0.0001). Transition between spectra indicated with gray shading. (C) Mean ± SEM changes in firing as a function of bar location for a representative MR unit in first 2 s (top) and last 2 s (bottom) of 10 s of “all-photoreceptor” (black) or “melanopsin-less” (orange) stimuli. Solid lines show Gaussian fits, R^2^ values shown top right. (D) Mean ± SEM firing rate (baseline subtracted) of MR (top) and non-MR units (lower panel; 18 and 51 units, respectively, recorded in 4 *Opn1mw*^R^ mice) at different contrast conditions for “all-photoreceptor” (black) or “melanopsin-less” (orange) stimuli. Michelson contrast for melanopsin is shown to right in black and the mean for rod and cone opsins in gray (%). Scale bar, 5 spikes/s. Transition between spectra indicated with gray shading. (E) A random binary modulation stimulus covering 0.1–15 Hz was generated and rendered in either “all-photoreceptor” or “melanopsin-less” stimuli. Top: example 6-s epoch of binary modulation stimulus. Bottom: power spectral density of the stimulus as a function of frequency. (F and G) Mean ± SEM cross power spectral density (CPSD) of MR (F) or non-MR units (G) in response to binary modulation stimulus rendered in “all-photoreceptor” (black) or “melanopsin-less” (orange) spectra. Significant differences in CPSD were found <0.88 Hz in MR (2-way ANOVA comparing single unit responses; significant effect of stimulus condition; p = 0.029. Post hoc comparison between frequencies: p < 0.01 at < 0.88 Hz) but not non-MR units (2-way ANOVA finds no effect of stimulus condition; p = 0.95). Grey bar indicates frequencies over which significant differences were detected. Inset of each panel shows unique melanopsin input to MR or non-MR units (“all-photoreceptor” – “melanopsin-less” response). (H) Histogram of the ratio of the integrated CPSD power from 0–1 Hz/1–2 Hz for individual MR (black, unfilled histogram) or non-MR units (gray filled histogram). Higher ratios indicate increased CPSD power at lower frequencies. (I) Circles represent the locations of a simulated RF superimposed onto a natural image; RF diameter approximates that of an individual MR unit (∼13°) and is scaled assuming that each image occupies a 180° field of view. The impact of small-moderate head and eye movements was simulated by shifting RFs randomly in space in movements of 2°–20° for 50 iterations (light blue circles). The impact of changes in gaze were then modeled by shifting the RF to a random location >40° away in the scene (dark blue circles). (J) The luminance in each RF shown in (I) (color code retained) as its location shifts according to the 50 simulated small-moderate eye movements and more substantial change in gaze. (K) The difference in luminance (Michelson contrast) encountered with each sequential movement of the RFs during the iterations shown in (I). (L) Probability distributions of the mean difference in luminance (Michelson contrast) over time; black distribution shows Michelson contrast for a population of MR unit RFs that tiled a number of natural images (n = 30) across 50 simulated iterations of small/moderate shifts in gaze (randomized movements <20°; simulated as in inset). Blue distribution shows Michelson contrast encountered for the same population of RFs following larger changes in gaze (random movement >40°; simulated as in inset for a population of MR unit RFs tiling 30 natural images). See also Figures S1–S3.

Having confirmed that melanopsin is sufficient to drive thalamic responses to spatial patterns, we next asked whether the representation of spatial patterns was deficient in the absence of melanopsin. The secondary impacts of melanopsin loss on visual function [10, 11, 13, 14, 15, 16, 17] make melanopsin-knockout mice unsuitable for answering this question. Thus, we adapted the silent substitution methodology to produce a third spectrally distinct input (spectrum 3) that enabled us to produce stimuli presenting equivalent contrast for rods and cones (33% long-wavelength sensitive [LWS], 23% short-wavelength sensitive [SWS], 33% rod opsin) but distinct melanopsin modulation (either 52% or <1%) depending on whether it was combined with spectrum 1 or 2 (Figure S1; Tables S1 and S2). These events are hereinafter termed “all-photoreceptor” and “melanopsin-less” stimuli, respectively (validation in Figures S1 and S3). We applied these spectra to the 10-s bar presentation paradigm in order to map spatial RFs (Figures 2A and 2B). RF locations and sizes of MR units were equivalent when mapped using either “all-photoreceptor” or “melanopsin-less” stimuli (Figures 2A and S3A). Responses under both conditions were characterized by a transient increase in firing that decayed over the 10 s for which the bar was present (Figure 2B). However, in MR units, the extent of decay was more substantial under the “melanopsin-less” condition (F test for exponential decay functions; p < 0.0001). This had the effect of reducing the amplitude of spatial RFs at late presentation times (Figures 2C and S3B). In non-MR units, responses to the two conditions were statistically indistinguishable (Figure S3C).

We adapted this approach to explore the contrast sensitivity of the melanopsin response, by generating matched “melanopsin-less” and “all-photoreceptor” stimuli over a range of melanopsin contrasts (Figures 2D and S3D). We found a significant difference in maintained firing in MR units at ∼43% (but not lower) Michelson contrast for melanopsin. This indicates that melanopsin makes a detectable contribution to the dLGN response to changes in radiance as small as 2.5-fold.

### Temporal Resolution of Melanopsin Responses

“Melanopsin only” responses were characterized by poor temporal resolution (Figure 1B), and this feature likely provides the primary constraint on melanopsin’s contribution to pattern vision, in which head/eye movements ensure that images falling on the retina are never stationary for long. We therefore next set out to directly measure melanopsin’s ability to track dynamic changes in light intensity. First, we recorded responses to a random binary modulation stimulus covering 0.1–15 Hz rendered in “melanopsin-less” versus “all-photoreceptor” conditions (Figure 2E) and used a cross power spectral density (CPSD) analysis between the stimulus and firing rate to quantify frequency tuning for single units (Figures 2F and 2G). A first observation was that the MR units had enhanced ability to track lower frequencies (<1 Hz) compared to non-MR units in the “all-photoreceptor” condition (Figure 2H). A further comparison between “melanopsin-less” and “all-photoreceptor” conditions revealed that this feature originates with melanopsin. Thus, CPSDs had reduced power at lower frequencies (<0.88 Hz) in MR (but not non-MR) units in the “melanopsin-less” condition (Figures 2F and 2G). These data thus indicate that melanopsin extends the temporal frequency range of dLGN vision (as previously proposed for the pupil light reflex [25]), allowing a subset of units to effectively track low-frequency (<∼1 Hz) modulations in light intensity.

### Melanopsin Responses to a Naturalistic Stimulus

**Figure 3:**
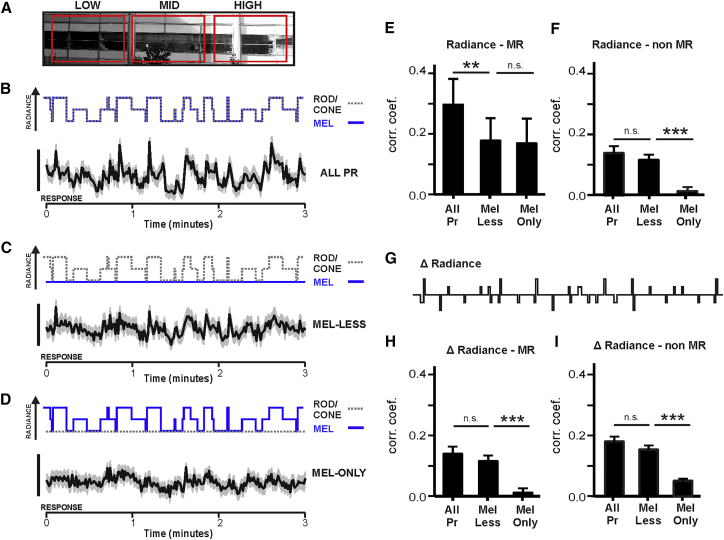
Melanopsin Helps Track Naturalistic Changes in Spatial Brightness (A) An image of a typical urban scene that contained regions of similar spatial contrast but differed in mean luminance (shown by bold red rectangles). To recreate natural viewing conditions of such a scene, a small region of view was extracted, and to account for common low-amplitude eye/head movements, was projected with an on-going jitter in position (frame shifted <10° at 4 Hz). (B–D) The projected image was rendered in different spectral mixtures over time to recreate shifting the field of view to regions of different shading: all-photoreceptors (B), melanopsin-less (C), or melanopsin-only (D). Top: changes in radiance for rods/cones (gray dashed line) or melanopsin (blue solid line), presented by transitions between stimuli occurring at intervals of 1, 5, or 10 s plotted over time. Bottom: mean ± SEM normalized firing rate of MR units (n = 10, recorded in three mice) to transitions between regions of different shading for each stimulus condition. Scale bar, 20% change in firing rate, relative to baseline. (E and F) The correlation between firing rate and stimulus radiance was calculated for MR (n = 10) (E) and non-MR (n = 84) (F) units in each stimulus condition (Pearson’s correlation coefficient comparing the firing rate of individual neurons and the normalized radiance of the stimulus). For MR units, RM one-way ANOVA revealed a significant effect of treatment p < 0.01 (post hoc Bonferroni test compared “melanopsin-less” with “all-photoreceptor” (^∗∗^p = 0.002) and “melanopsin-only” (p = 0.99). For non-MR units, RM one-way ANOVA revealed a significant effect of treatment p < 0.01 (post hoc Bonferroni test compared “melanopsin-less” with “all-photoreceptor” (p = 0.16) and “melanopsin-only” (^∗∗∗^p < 0.001). (G) Schematic of the change in stimulus output over time (Δ radiance). (H and I) The correlation between firing rate and the change in stimulus was calculated for MR (n = 10) (H) and non-MR (n = 84) (I) units in each stimulus condition (Pearson’s correlation coefficient comparing the firing rate of individual neurons and the normalized Δ radiance of the stimulus). For MR units, RM one-way ANOVA revealed a significant effect of treatment p < 0.01 (post hoc Bonferroni test compared “melanopsin-less” with “all-photoreceptor” (p = 0.9) and “melanopsin-only” (^∗∗∗^p = 0.006). For non-MR units, RM one-way ANOVA revealed a significant effect of treatment p < 0.01 (post hoc Bonferroni test compared “melanopsin-less” with “all-photoreceptor” (p = 0.11) and “melanopsin-only” (^∗∗∗^p < 0.004). See also Figure S3.

How might these characteristics allow melanopsin to augment form vision under more natural conditions? When viewing a stationary scene, changes in radiance within RFs of individual units originate with changes in direction of view. An inverse relationship between the magnitude and frequency of head and eye movements has been described in numerous species, including nocturnal rodents [29, 30, 31, 32]. As natural scenes show strong correlations in local radiance [33, 34], light falling within an individual RF may therefore be relatively invariant across small shifts in gaze, while larger magnitude shifts present more substantial contrasts (see demonstration in Figures 2I–2L). The results above indicate that melanopsin may contribute to maintaining the representation of patterns under such conditions. We produced a stimulus to test this prediction based on a natural scene in which a fine spatial scale pattern is repeated under different levels of shade (Figure 3A). We presented this scene to anesthetized mice and recreated the effect of frequent, small amplitude changes in direction of view using ongoing 4 Hz shifts in its phase, randomized for magnitude (<10°, mean = 7.8°) and direction. We rendered the low spatial frequency pattern in shade visible either to melanopsin alone, rods and cones alone, or all photoreceptors using the “melanopsin-only,” “melanopsin-less,” or “all-photoreceptor” stimulus pairs defined above. The impact of the larger, less frequent changes in gaze was produced by shifting the presented image across this pattern of shade (Figures 3B–3D).

We recorded dLGN activity in response to the presentation of a movie which included these features (Figures 3B–3D). We then computed the Pearson’s correlation coefficient between firing rate of individual dLGN units and the simulated shade of the image in the field of view (Figures 3E and 3F). As expected, the correlation coefficient of non-MR units was equivalent for “all-photoreceptor” and “melanopsin-less” versions of the stimulus and close to zero (mean ± SEM: 0.012 ± 0.014) under the “melanopsin-only” condition (Figure 3F). By contrast, in MR units (Figure 3E), correlation coefficients were significantly larger when the changes in shade were visible to all photoreceptors than only rods and cones and equivalent for “melanopsin-less” and “melanopsin-only” stimuli. These data indicate that melanopsin enhances the ability of MR units to encode maintained differences in local radiance. An equivalent assessment of correlation was made between firing rate and the instantaneous change in radiance (that we expect to be adequately tracked by rods and cones; Figures 3G–3I). In this case, correlations were similar in “all-photoreceptor” and “melanopsin-less” conditions for MR and non-MR units but close to zero for the “melanopsin-only” condition.

### Predicting Melanopsin Contributions under Active View

**Figure 4:**
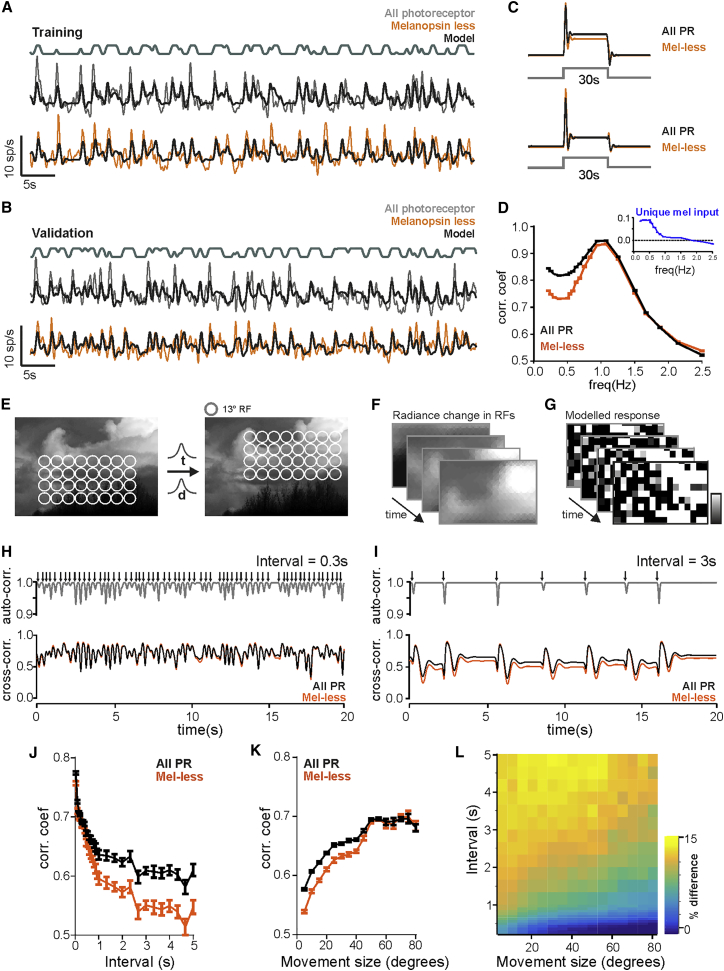
Modeling Melanopsin’s Contribution to Spatial Vision (A) Responses to the binary modulation stimulus rendered in “all-photoreceptor” or “melanopsin-less” stimuli (mean responses of 11 MR units [from 56 units recorded in five animals] for a 60-s epoch shown with gray and orange lines, respectively) were fitted using an autoregressive exogenous (ARX) model (thick black line). Above gray line shows model input (smoothed stimulus). (B) Models were validated on a separate response epoch to the binary modulation stimulus (mean responses for a 60-s epoch shown with gray and orange lines, for “all-photoreceptor” and “melanopsin-less,” respectively; thick black line shows model prediction). Above gray line shows model input (smoothed stimulus). (C) Model-predicted responses to a 30 light step rendered in “all-photoreceptor” (black) and “melanopsin-less” (orange) for MR units (top) and non-MR units (bottom). Stimulus presentation depicted with gray lines. (D) Mean ± SEM correlation between stimulus and modeled responses generated for “all-photoreceptor” (black) and “melanopsin-less” (orange) conditions for a range of binary modulation stimuli spanning different frequency ranges (summarized in Figure S3F; repeated for 25 modeled neurons). Correlation coefficients were significantly different at frequencies <1 Hz. Inset: unique melanopsin input (“all-photoreceptor” – “melanopsin-less” response). (E–G) Cartoon depicting the process of modeling MR unit responses over space and time. Natural images were tiled with an array of MR unit RFs (E) (13° diameter). The array of MR RFs was then moved across the natural image with a range of intervals (*t*) and distances (*d*). The image was filtered through the overlapping array of modeled RFs (F). The correlation coefficient between stimulus and the modeled responses (G) of an array of neurons tiling the natural image was then calculated for each frame in a 30-s epoch. (H and I) Images were moved across an array of RFs (mean distance = 30°) for mean intervals of 0.3 s (H) and 3 s (I). Arrows depict movement events over a 20-s epoch. Top: spatial autocorrelation between frames (low correlation occurring following image movement). Bottom: spatial cross-correlation between stimulus and modeled responses for each frame over time (“all-photoreceptor” and “melanopsin-less” linear filters depicted with black and orange lines respectively). Note elevated cross-correlation in “all-photoreceptor” model for periods of high auto-correlation in (I). (J) Mean ± SEM correlation coefficient for a range of intervals (mean interval between movement of 0.03–5 s), for a fixed movement size (mean ± SD 10° ± 3°), for “all-photoreceptor” and “melanopsin-less” models (black and orange lines, respectively). Significant differences in correlation were found at intervals >0.4 s). (K) Mean ± SEM correlation coefficient for a range of movement sizes (mean amplitudes of 5°–80°), for a fixed inter-stimulus interval (mean ± SD 1 ± 0.3 s), for “all-photoreceptor” and “melanopsin-less” models (black and orange lines, respectively). Significant differences in correlation were found at movement sizes <45°). (L) Heatmap showing % difference in correlation values generated for “all-photoreceptor” and “melanopsin-less” responses modeled across a range of intervals (0.03–5 s) and amplitudes (5°–80°). See also Figure S3.

We finally set out to provide a more general framework for predicting melanopsin’s contribution to encoding images under natural view. To begin, we used dLGN responses to the binary noise stimulus to define linear mathematical functions predicting single unit firing in the presence and absence of melanopsin activity. Using a linear ARX model (see the STAR Methods), we determined optimal parameter settings for MR units during a training epoch for “all-photoreceptor” and “melanopsin-less” stimuli (Figure 4A; Pearson’s correlation coefficient = 0.68 and 0.65, respectively) and confirmed their suitability against a separate validation epoch (Figure 4B; Pearson’s correlation coefficient = 0.67 and 0.64 for “all-photoreceptor” and “melanopsin-less” conditions, respectively). The models constructed for “melanopsin-less” and “all-photoreceptor” conditions captured melanopsin’s contribution to encoding steady (Figure 4C) and slowly changing light (frequencies <1 Hz; Figures 4D and S3F). Using these “all-photoreceptor” and “melanopsin-less” linear filters, we were also able to adequately describe the activity of individual dLGN units over time under the simulated natural view experiment (Figure S3G).

We applied these models to a simulated group of MR units tiling the visual scene in order to predict the representation of spatial patterns by the dLGN population under different viewing conditions. We took a library of calibrated natural images (n = 30) and tiled each with an array of RFs matching those of MR units (13° 2D Gaussians) and recreated shifts in direction of view over a range of frequencies and magnitudes (Figures 4E–4G). We then calculated the mean radiance falling within each RF over time and applied “all-photoreceptor” and “melanopsin-less” linear temporal filters to predict the activity profile of the underlying MR neuron under conditions in which spatial information was available from all photoreceptors or just rods and cones. We finally calculated the cross-correlation over time between the spatial pattern in radiance and the modeled array of responses as a simple metric for the degree to which the scene was represented in the firing pattern of dLGN neurons (see representative examples in Figures 4H and 4I). We found that this correlation was higher for the “all-photoreceptor” simulation over a wide range of conditions. Consistent with the low temporal resolution of melanopsin vision, the “melanopsin-less” correlation was most deficient when the stimulus simulated less frequent or lower amplitude changes in direction of view (Figures 4J–4L).

## Discussion

Developing a bespoke, 5-primary visual display has provided us with a unique opportunity to quantify the spatiotemporal resolution of melanopsin vision. In generating visual images that provide spatiotemporal contrast to rods and cones versus melanopsin, we have shown that melanopsin signals have sufficient spatiotemporal resolution to encode spatial patterns. Our description of melanopsin spatial RFs in the mouse dLGN show that its spatial resolution is modest (RF diameters ∼13°), but that is true for mouse vision in general, and this feature is likely to be species-specific. Thus, the melanopsin RFs we observe are approximately equivalent to those of afferent ipRGCs (estimated RF diameter ∼6°–17° based on anatomical and electrophysiological data [12, 35, 36]), implying that they are defined by ipRGC dendritic architecture and the size of the mouse eye. Melanopsin spatial RFs could therefore be significantly smaller in other species, including humans, in which the dendritic field of individual ipRGCs occupies a smaller fraction of visual space (predicted RF diameters ∼1.5°–3° in humans [37]).

Perhaps the primary limitation on melanopsin’s contribution to encoding spatial patterns is its temporal response properties. Our findings are consistent with the established view that melanopsin’s particular contribution to mammalian vision is its ability to track steady and slowly changing light. These have previously been considered in relation to the problem of tracking ambient light. However, by carefully quantifying this characteristic, our data reveal that melanopsin has the temporal resolution also to contribute directly to form vision. Coarse patterns are common in natural images [33, 34], and there is an inverse relationship between the frequency and magnitude of changes in gaze in all species examined [29, 30, 31, 32]. It follows that radiance within individual RFs varies over multiple timescales and can be relatively invariant for extended periods. To relate the activity recorded here to this aspect of active viewing, we have created linear filters allowing the firing pattern of individual units in the presence and absence of melanopsin input to be predicted. Applying these models to natural scenes filtered according to the melanopsin spatial RF predicts that, indeed, melanopsin can augment the thalamic representation of natural images. The magnitude of this contribution is largest in epochs during which changes in gaze are small in magnitude and/or relatively infrequent. The range of conditions in which melanopsin could be relevant is, however, quite wide and not restricted to strict fixation, with a contribution to representing natural scenes predicted even with changes in view as large as 40° and as frequent as once per second in the example used here.

Although the absolute magnitude of melanopsin-evoked changes in firing we observe is modest (∼1 Hz), this figure needs to be viewed with caution. Our experiments employ anesthetized mice to allow us to present carefully calibrated stimuli over long durations. Although anesthesia does not alter the relative response of visual neurons to different types of stimuli, it does induce a general suppression of baseline and visually evoked firing [11, 38]. Therefore, it is most unlikely that the melanopsin response is equivalently small in awake animals. Better frames of reference are the responses we record to other visual stimuli (roughly equivalent to response evoked by 2 Hz inverting checker; Figure 1F), response magnitude in relation to baseline activity (∼30% increase; Figures 1 and S3), and the fraction of the total visual response attributable to melanopsin (∼40% correlation coefficient between firing and local radiance for simulated natural view; Figure 3).

How might the melanopsin signal contribute to perception and pattern discrimination? One possibility is that melanopsin enhances the appearance of spatial contrast across larger areas of the visual scene. Perceiving coarse patterns has previously been explained in terms of higher visual processes of surface interpolation and filling in [39]. The melanopsin signal we describe here might contribute to these processes. Increases in melanopsin excitation induce percepts of “brightness” in humans and influence brightness discrimination in behavioral tasks in mice [19, 20]. Our data suggest that this melanopsin “brightness” percept might extend to distinguishing patterns.

Our data have implications for methods of image capture and display. Current technology relies on the red-green-blue (RGB) additive color model, which exploits the observation that three separately modulated, spectrally distinct, inputs (“primaries”) can be used to gain control of the human three cone photoreceptors and recreate perceptions of color (chroma and saturation) and brightness (luminance). Our data imply that appropriate representation of visual scenes should also take account of melanopsin. Including an additional primary in display devices would allow control over the spatial pattern of melanopic radiance, creating more natural representations of visual scenes.

## STAR★Methods

### Key Resources Table

**Table:** 

REAGENT or RESOURCE	SOURCE	IDENTIFIER
**Experimental Models: Organisms/Strains**		

*Opn1mw*^*R*^	Jeremy Nathans [40]	RRID:MGI:2678771
*Opn1mw*^*R*^; *Opn4*^−/−^	Jeremy Nathans [40]	RRID:MGI:5694632
	King-Wai Yau [41]	

**Software and Algorithms**		

MATLAB R2015a	The Mathworks	https://www.mathworks.com/products/matlab.html
Prism 7	GraphPad Software	https://www.graphpad.com/scientific-software/prism/
Neuroexplorer	Nex Technologies	http://www.neuroexplorer.com/
Offline Sorter	Plexon	http://www.plexon.com/products/offline-sorter
LabView 8.6	National Instruments, Ltd	http://www.ni.com/labview/
Python	Python Software Foundation	https://www.python.org/
PsychoPy	Jonathan Peirce	http://www.psychopy.org/
Arduino	Arduino	https://www.arduino.cc/
ChipKit	MPIDE	http://chipkit.net/tag/mpide/

### Contact for Reagent and Resource Sharing

Further information and requests for resources, reagents, or raw data should be directed to and will be fulfilled by the Lead Contact, Annette Allen (annette.allen@manchester.ac.uk)

### Experimental Model and Subject Details

#### Animals

Experiments were performed on *Opn1mw*^*R*^ and *Opn4*^*−/−*^*; Opn1mw*^*R*^ male mice (aged 3-6 months) from a C57/BL6 background. *Opn1mw*^*R*^ refers to the transgenic allele originally generated by Smallwood et al. (2003), and termed “R” by them [40]. *Opn4*^*−/−*^ mice contain an insertion of tau-lacZ into the melanopsin gene locus [41], rendering mice ‘melanopsin-knockout’ [17]. Animals were kept in a 12 hr dark/light cycle at a temperature of 22°C with food and water available ad libitum. All animal care was in accordance with the Animals, Scientific Procedures, Act of 1986 (UK), and approved by the local (The University of Manchester) ethics committee. Experimental animals were randomly selected from a large colony.

### Method Details

#### In vivo physiology

Anesthesia was induced with an intra-peritoneal injection of urethane (1.6g/kg; 30%w/v; Sigma-Aldrich). A topical midriatic (1% (w/v) atropine sulfate; Sigma-Aldrich) and mineral oil (Sigma-Aldrich) were applied to the left eye prior to recordings. After placement into a stereotaxic frame, the mouse’s skull surface was exposed and a small hole drilled ∼2.3mm posterior and ∼2.3mm lateral to the bregma. A recording probe (A4x8-5mm-50-200-413; Neuronexus) consisting of 4 shanks spaced 200μm apart, each with 8 recording sites (spaced 50μm, sized 413μm^2^), was lowered a depth of ∼2.5-3mm into the brain, targeting the dorsal lateral geniculate nucleus (dLGN). Neural signals were acquired using a Recorder64 system (Plexon), and were amplified (x3000), highpass filtered (300Hz), and digitised at 40kHz. Multiunit activity was saved and analyzed offline using Offline Sorter (Plexon). After removing artifacts common to all channels, single-unit spikes were detected and categorized based on the spike waveform via a principal component analysis, whereby distinct clusters of spikes were readily identifiable, that showed a clear refractory period in their interspike interval distribution. In addition, Isolation distances were quantified for all isolated units (distribution in Figure S4), and a threshold of an isolation distance > 50 was exceeded in all but 6 isolated units. Spike sorted data were then further analyzed using Neuroexplorer (Nex Technologies) and MATLAB R2015a (The Mathworks), to assess the changes in firing rate of neurons in response to different visual stimuli.

#### Histology

To establish the location of recording sites, the recording electrode was dipped in fluorescent dye (Cell Tracker CM-DiI; Invitrogen) prior to insertion. In other experiments we have found good correspondence between electrode placements reconstructed using this method and by use of electrolytic lesions. Following recordings, the mouse’s brain was removed and post-fixed overnight in 4% paraformaldehyde, prior to cryoprotection for 24 hr in 30% sucrose. 99 μm coronal sections were then cut using a sledge microtome, mounted onto glass slides and coverslips were applied using Vectashield (Vector Laboratories).

#### Visual stimuli

Structured images were presented using a custom-made light source containing four independently controlled LEDs (λ_max_ and full width half max (FWHM): 405nm (FWHM = 15nm), 455nm (FWHM = 10nm), 525nm (FWHM = 25nm), 630nm (FWHM = 15nm); Phlatlight PT-120 Series (Luminus Devices)) and a laser (GEM 561nm; 500mW; Laser Quantum, UK). Light from the LEDs was combined by a series of dichroic mirrors (Thorlabs), and directed into a digital mirror device (DMD) projector (DLP® LightCommander; Logic PD) in place of the original intrinsic light source.

LED intensities were controlled with two Micro-controllers (chipKit UNO32 Digilent, WA, USA, and Arduino Due; Arduino) and associated software (MPIDE and Arduino IDE). The R G and B input channels of the DMD projector were then synchronized to each display a distinct combination of our five channels, allowing us to present spatial patterns using these five different wavelengths. The LEDs and laser were combined to generate three background and stimulus combinations that are summarized in Figure S1 and in Tables S1 and S2. Dynamic spatial and temporal changes in the presentation of these spectra were then generated using Python running PsychoPy Version 1.70.00 [42].

To expand the area of the retina exposed to our spatial stimuli, additional LED lighting surrounded the projection screen. These LEDs (peak emission at 400nm (Component-Shop), 460nm, 517nm and 630nm (LEDLightsZone)) were arranged in a high-density array, and were placed behind Opal Polypropylene (2mm thickness; The Plastic People) to create a diffuse surround. LED intensities were controlled with a PC running LabView 8.6 (National Instruments) and matched equivalent photon fluxes of the projection screen.

##### Light calibration

Stimuli were measured at the corneal plane using a spectroradiometer (Bentham Instruments) between 300-800nm. The effective photon flux for each photopigment was then calculated by weighting spectral irradiance according to pigment spectral efficiency profile as estimated by the pigment spectral efficiency function (derived from a visual pigment template [43] and λ_max_ values of 365, 480, 498, 508 and 556nm for SWS opsin, melanopsin, rod opsin, MWS opsin and the introduced LWS opsin respectively) mutliplied by an in vivo measurement of spectral lens transmission [44]. The approach is equivalent to that described in [45], using spectral efficiency functions available at: http://lucasgroup.lab.ls.manchester.ac.uk/research/measuringmelanopicilluminance. Though other opsins are present within the murine retina [46, 47], electrophysiological recordings from retina and brain show that animals lacking all known photopigments show no evidence of these opsins driving changes in firing rate [48, 49].

##### Identification of light responses

In all cases, responses were classed as light-responsive if the firing rate during stimulus presentation exceeded baseline firing rate by more than 2 standard deviations of the mean baseline firing rate (prior to light exposure).

##### Full-field stimuli and online calibration

In all mice (n = 25), full field flashes (4Hz; 50 repeats) or steps (10 s steps with 60 s inter-step interval; 10 repeats) between stimulus spectra were used to calibrate and validate each stimulus condition. In all experiments, we began with a phase of online calibration, in which we isolated the particular stimulus settings which failed to produce rod/cone responses (but which drove significant melanopsin contrast). To do this, fast transitions between two spectral combinations were presented on the entire screen to the mouse (4Hz). The stimulus spectra was adjusted every 50 repeats, such that the output of the blue LED changed in ∼7% steps. By decreasing and increasing the intensity of the blue LED around our calculated stimulus, we were able to generate flash responses either side of the estimated rod/cone isoluminant point, until the setting was such that no responses were measurable (see Figure S1L for representative unit). In all cases, the blue intensity identified as physiological silent for rod/cone responses was within ± 7% of our empirically predicted setting.

##### Receptive field mapping

Vertical bars (occupying ∼13° of the visual field) were used to map horizontal RFs of dLGN neurons. Bars were presented in a pseudorandom order in 13 (overlapping) spatial locations (4.5° separation in bar position). We found this stimulus regime drove equivalent receptive fields to a higher-resolution protocol (Figures S2D–S2F). To identify melanopsin-driven responses, the spectra of background and bars were those described above for ‘all-photoreceptor’, ‘melanopsin-only’ or ‘melanopsin-less’ (Figure S1). Bars were presented in a pseudo-randomized sequence for 10 s, with an inter-stimulus interval of 60 s. For melanopsin-only RFs (n = 6 mice), this protocol was repeated 30 times. For ‘all-photoreceptor’ and ‘melanopsin-less’ RFs (n = 6 mice), this protocol was repeated 15 times in each condition in an interleaved manner. In all mice (n = 25), an equivalent high-speed RF mapping protocol was also used to map conventional RFs, in which bars were presented for 50ms with an inter-stimulus interval of 250ms, using the ‘all-photoreceptor’ stimulus. Spatial RFs were then derived from the responses to this sequence. Spatial RF sizes were estimated in the horizontal dimension by fitting a Gaussian to the responses evoked by bars covering discrete parts of visual space. The RF size for individual cells was described as the standard deviation of a Gaussian fitted to each dimension. To compare whether spatial RFs differed between conditions, in each dimension, Gaussians were compared with an F-test, to test whether RFs were best fit with a single, or two individual Gaussians.

##### Contrast responses

In a separate set of experiments, we modified the presentation of pairs of stimuli by making the stimulus a ratio of background and stimulus spectra; for example, to present our ‘all-photoreceptor’ stimulus with *half* the effective contrast, we transitioned from background spectrum A to a 50/50 mix of spectrum A and spectrum C. This allowed us reduce the contrast steps presented by the ‘all-photoreceptor’ and ‘melanopsin-less’ stimuli. These additional stimuli were all fully calibrated. We recorded the responses these stimuli, presented across the entirety of our projection screen and in an interleaved fashion (10 s stimulus; 30 s inter-stimulus interval) in four *Opn1mw*^*R*^ and three *Opn1mw*^*R*^*; Opn4*^*−/−*^ mice.

##### Inverting chequerboards

In three mice, inverting chequerboard stimuli (7.5° squares; 3-fold change in radiance between dark and light squares) were presented with an inversion every 250ms, in which light and dark squares were high- and low-energy versions of spectrum 1. A change in spectrum (either spectrally neutral change, or a transition from spectrum 1 to spectrum 2) was introduced to a large area of the screen (∼33°, in one of two locations) for 10 s (inter-stimulus interval of 30 s) while maintaining presentation of chequerboards of an equivalent contrast. This was repeated 10 times.

##### Binary modulation stimulus

In 6 mice, full field square-wave transitions between pairs of stimuli were presented in a pseudorandom sequence. Transitions covered a range of frequencies (0.1-15Hz; Figure 2E), and the same temporal sequence was presented for a period of 15 min either between spectrum 1 to 3 (‘all photoreceptors’) or 2 to 3 (‘melanopsin-less’) with a randomized order. The cross power spectral density was then calculated to provide a measure of correlation between stimulus and response as a function of frequency.

##### Autoregressive model

Responses to the binary modulation stimulus were generated by using a linear autoregressive model with exogenous input (ARX). The parameters of the model were estimate by fitting the mean response (discretized into 33ms time bins) of MR and non-MR units in response to binary modulation stimuli rendered in ‘all-photoreceptor’ or ‘melanopsin-less’ spectra. To capture the main features of these responses the model included 5 inputs and 2 autoregressive terms. The inputs were represented by rectified positive and negative, background subtracted, radiance (kept separated to account for differences in ON and OFF pathways), their derivatives, and a constant term to adjust the basal firing rate. To account for photoreceptor and inner retinal smoothing of the visual signal all inputs were also smoothed by using a box-car filter (100ms duration).

To ensure that neurons with robust responses were included in further analyses, the value of Pearson’s correlation between the mean response of individual neurons and their modeled response was compared with values obtained after shuffling the mean response across time bins (1000 repeats); only those neurons whose correlation exceeded 2 standard deviations of shuffled data were included in further analyses (56/197 light responsive neurons). The population response was then obtained by averaging across those neurons and used to estimate the linear ARX models with ‘all-photoreceptor’ and ‘melanopsin-less’ stimuli. The goodness of fit for these models was assessed using Pearsons’s correlation. The models were fit by using 15 min recordings which were split in into 1 min epochs. Two sets of interleaved epochs were then used for training and validation of the models (Pearson's correlation > 0.5).

The linear models generated for ‘all-photoreceptor’ or ‘melanopsin-less’ were subsequently used to predict responses to other temporally and spatially modulated stimuli. A range of binary noise stimuli were generated that spanned different frequency ranges (0.1 to 10Hz; standard deviation of frequency distributions equivalent to mean/3). Natural images were also used to combine spatial and temporal models of responses. 100 images were sampled from a natural image database [50], onto which a grid of MR unit receptive fields was mapped (RF size = 13°). Since natural scenes are scale invariant [34], in our hands we assumed each image to occupy 180x120°. These images were then shifted randomly in x and y coordinates in a distance and direction sampled from a distribution *d*, and with an interval sampled from the distribution *t.* The mean radiance falling within each RF over time was calculated and the ‘all-photoreceptor’ and ‘melanopsin-less’ linear temporal filters were used to predict the activity profile of each MR neuron. Pearson’s correlation coefficient was then calculated between the spatial pattern in radiance and the modeled array of responses over time. The distributions of *d* and *t* were adjusted to explore how melanopsin makes a contribution to visual scenes during different eye movements.

##### Naturalistic viewing

In three mice, an image of a typical urban scene (windows on a Manchester building) was displayed at bright, mid, and dim luminance levels for 1 s, 5 s, or 10 s, in a pseudo-random sequence lasting 3 min, for ‘all-photoreceptor’, ‘melanopsin-less’, or ‘melanopsin-only’ stimulus spectra, and was repeated 10 times. This image was presented with an on-going jitter in position (frame shifting < 10 degrees at 4Hz) in order to recreate eye movements. Responses to low-frequency changes in luminance were analyzed by calculating Pearson’s correlation coefficient between firing rate of dLGN units and the global effective radiance of the scene.

##### Natural image analysis

We superimposed an array of circular RFs, matching the mean RF size of MR dLGN neurons (∼13°) onto 30 black and white images randomly selected from an open access database [50] that were assumed to occupy 180°. These RFs were then moved random distances 2-20° in any direction for 50 iterations. We calculated the mean radiance within each RF for each of these locations, and the time-averaged radiance of each RF. The impact of changes in gaze were then modeled by shifting the RF to a random location > 40° away in the scene (dark blue circles). We quantified the Michelson contrast between all pairs of RFs over time, and calculated the probability distribution of contrast occurring during small (< 20°) or large (> 40°) movements across the population of natural images.

### Quantification and Statistical Analysis

All statistical analyses were performed using MATLAB R2015a (The Mathworks) or Prism 7 (GraphPad Software). Data throughout the manuscript are presented mean ± SEM, unless otherwise stated in Figure legends. Details of n for each experiment (n = number of animals, and n = numbers of units), and the particular statistical tests used, can be found within Figure legends. Parametric tests were used throughout to compare responses to the above stimuli rendered in different stimulus spectra (‘all-photoreceptor’, ‘melanopsin-less’, ‘melanopsin-only’). Repeated-measures analyses were used when comparing responses of individual units to ‘all-photoreceptor’ versus ‘melanopsin-less’ stimuli, and when comparing receptive fields mapped with ‘all-photoreceptor’ versus ‘melanopsin-only’ stimuli. Repeated-measures ANOVAs (with a Bonferonni correction applied for post hoc multiple comparisons) were used to examine responses of single units to RF mapping over time, and to compare correlation coefficients computed during the *Naturalistic-viewing* protocol. A p value of 0.05 was used to define significance. In post hoc analyses, any unit that showed a significant modulation in firing rate (see above ‘*Identification of light-responses’*) in response to 4Hz transitions between the final calibrated melanopsin-only stimulus was excluded from subsequent analyses. In all RF mapping procedures, any unit whose peak response occurred at the screen edge was excluded due to receptive field ambiguity.

## Author Contributions

Conceptualization, A.E.A. and R.J.L.; Methodology, A.E.A. and F.P.M.; Formal Analysis, A.E.A. and R.S.; Investigation, A.E.A., R.A.B., and R.S.; Writing – Original Draft, A.E.A. and R.J.L.; Writing – Review & Editing, A.E.A., R.J.L., and RS; Funding Acquisition, R.J.L.

## Supplemental Information

Document S1. Figures S1–S4 and Tables S1–S2

Document S2. Article plus Supplemental Information
