# Supplementary material for: Melanopsin Contributions to the Representation of Images in the Early Visual System
Source: Curr Biol. 2017 Jun 5;27(11):1623–1632.e4. doi: 10.1016/j.cub.2017.04.046 (PMC5462620; doi:10.1016/j.cub.2017.04.046)
Supplement: Document S1. Figures S1–S4 and Tables S1–S2 [file mmc1.pdf]

Current Biology, Volume *27*

Supplemental Information

Melanopsin Contributions to the Representation of Images in the Early Visual System

Annette E. Allen, Riccardo Storchi, Franck P. Martial, Robert A. Bedford, and Robert J. Lucas

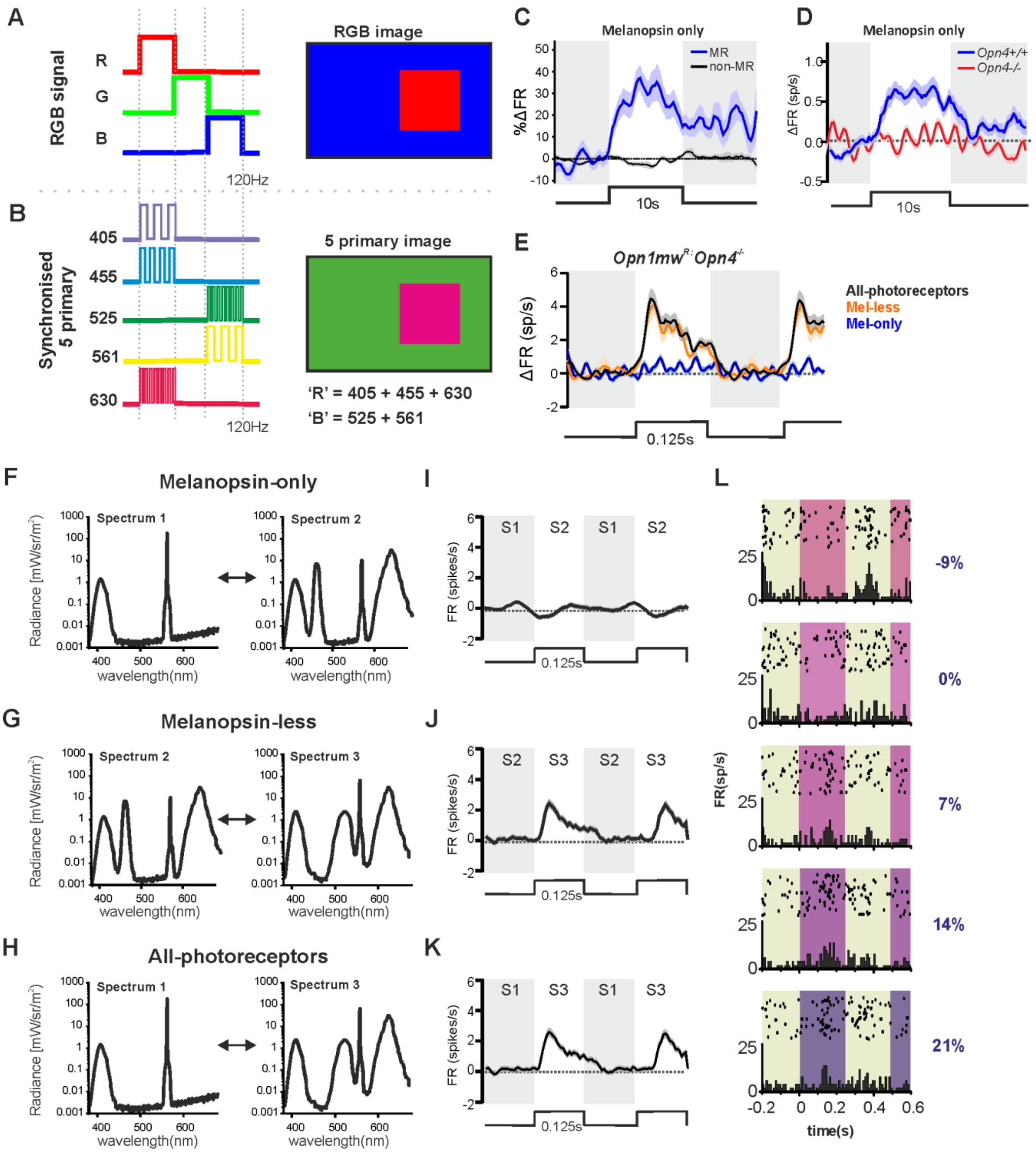

Figure S1. Design, calibration and validation of stimuli; Related to Figures 1 and 2.

A, In order to generate spatial patterns with up to 5 primary inputs, each colour plane of an image (Red, Green, or Blue) is separated in time (120Hz; left panel).

B, A microcontroller sends five PWM values to the LED drivers that are synchronised in time to each colour plane (a simple example shown in left panel of **B**). In this way, any combination of the 5 primaries may be assigned to each colour plane, thus generating a 5 primary image (e.g. right panel of **A&B**).

C, Firing rate as a % of pre-stimulus firing in response to a 10s presentation of 'melanopsin-only' stimulus every 60s (i.e. 10s presentation of spectrum 2 interleaved with a background of spectrum 1). Data shows responses from 166 MR (blue line) and 502 non-MR units (black line) recorded in 26 *Opn1mw*^{R} mice. Timing of stimulus shown below as step and as an interruption of shading on main plot. Dotted line shows 0% change in activity.

D, Mean (± SEM) firing rate (baseline subtracted) in response to a 10s presentation of 'melanopsin-only' stimulus every 60s. Data shows responses from 110 light-responsive units recorded in 6 *Opn1mw*^{R}*; Opn4*$^{-/-}$ mice (red line), and 166/668 units recorded in 26 *Opn1mw*^{R} mice (blue line). Timing of stimulus shown below as step and as an interruption of shading on main plot. Dotted line shows baseline activity.

E, Mean (± SEM) firing rate (baseline subtracted) of 110 light responsive units recorded in 6 *Opn1mw*^{R}*; Opn4*$^{-/-}$ mice in response to 4Hz transitions between spectra 1 and 3 ('all-photoreceptor'; black line); 2 and 3 ('melanopsin-less'; orange line), or 1 and 2 ('melanopsin-only'; blue line). The mean response of the 105 light-responsive units showed no difference in their response amplitude to 'all-photoreceptor' and 'melanopsin-less' stimuli (paired two-tailed T-test of peak response: P = 0.13). 3/110 individual units crossed our threshold of 2-standard deviations above baseline firing, though their response was qualitatively different from that in the melanopsin-sufficient condition.

F-H, The output of four LEDs (peak emissions: 405nm, 455nm, 525nm, 630nm) and a 561nm laser were controlled to produce three spectra (spectra 1-3). Transitions between these three spectra were calculated to provide contrast for melanopsin in isolation (**F**; termed 'melanopsin-only'; from spectrum 1 to 2), or rod and cone opsins (**G**; termed 'melanopsin-less'; from spectrum 2 to 3). Transitions from spectrum 1 to 3 (**H**; termed 'All-photoreceptor') drove the sum of the contrasts from 'melanopsin-only' and 'melanopsin-less' (i.e. the same contrasts provided for melanopsin, or rods and cones in the 'melanopsin-only' or 'melanopsin-less' conditions, respectively).

I-K, Mean (± SEM) firing rate (baseline subtracted) of 166/668 units (those that responded to a 10s 'melanopsin-only' stimulus) recorded in *Opn1mw*^{R} mice in response to 4Hz transitions between spectra 1 and 2 (**I**; 'melanopsin-only'), 2 and 3 (**J**; 'melanopsin-less'), and 1 and 3 (**K**; 'all-photoreceptor'; black line);

L, Response of one representative unit to the online-calibration of our 'melanopsin-only' stimulus, during which the relative power of the 455nm LED in spectrum 2 was adjusted. Graphs show raster plots of 50 repeats of stimuli, with mean PSTH plotted below. Numbers to right of plots show percentage change from calibrated 455nm power. Transitions between spectra were projected onto a screen occupying 57x72° of visual space, surrounded by a poly-spectral uniform array of LEDs that were evenly diffused. Shifting from low to higher values resulted in rod/cone responses occurring during spectrum 1 (OFF response) to spectrum 2 (ON response).

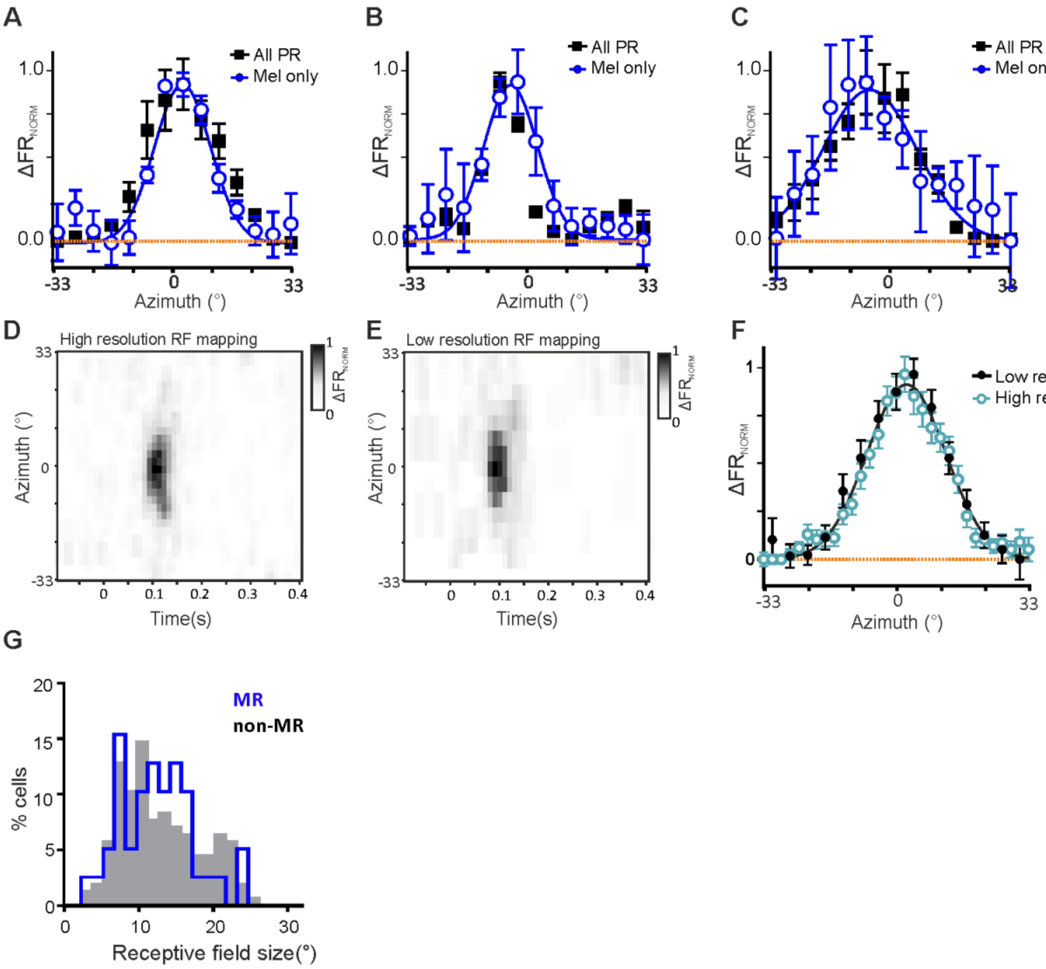

Figure S2. Receptive field mapping comparisons and representative examples; Related to Figures 1 and 2 and STAR Methods.

A-C, Three further representative MR unit responses to the melanopsin-only receptive field mapping protocol (blue; using 13° bars present for 10s with 60s interval), and all-photoreceptor receptive fields (black; using 13° bars present for 250ms every 2.5s). Data shows mean±SEM normalised response amplitude at time-point eliciting maximum response as a function of bar position). Orange dotted line shows baseline activity level.

D&E, Change in firing rate of a representative unit as a function of location on the azimuth using high (**D,** 13° bars) and low-resolution (**E,** 6° bars) receptive field mapping protocols. Plots show change in firing over time as heat map (scale to right).

F, Spatial receptive fields (mean±SEM normalised response amplitude at time-point eliciting maximum response) of data from **D&E**, for high and low-resolution mapping (black and cyan circles, respectively). Data are best fit with a single Gaussian curve (F-test comparison; P>0.05). Orange dotted line shows baseline activity level.

G, Histogram of receptive field sizes of MR and non-MR units, mapped using an all-photoreceptor stimulus (13° bars present for 250ms every 2.5s).

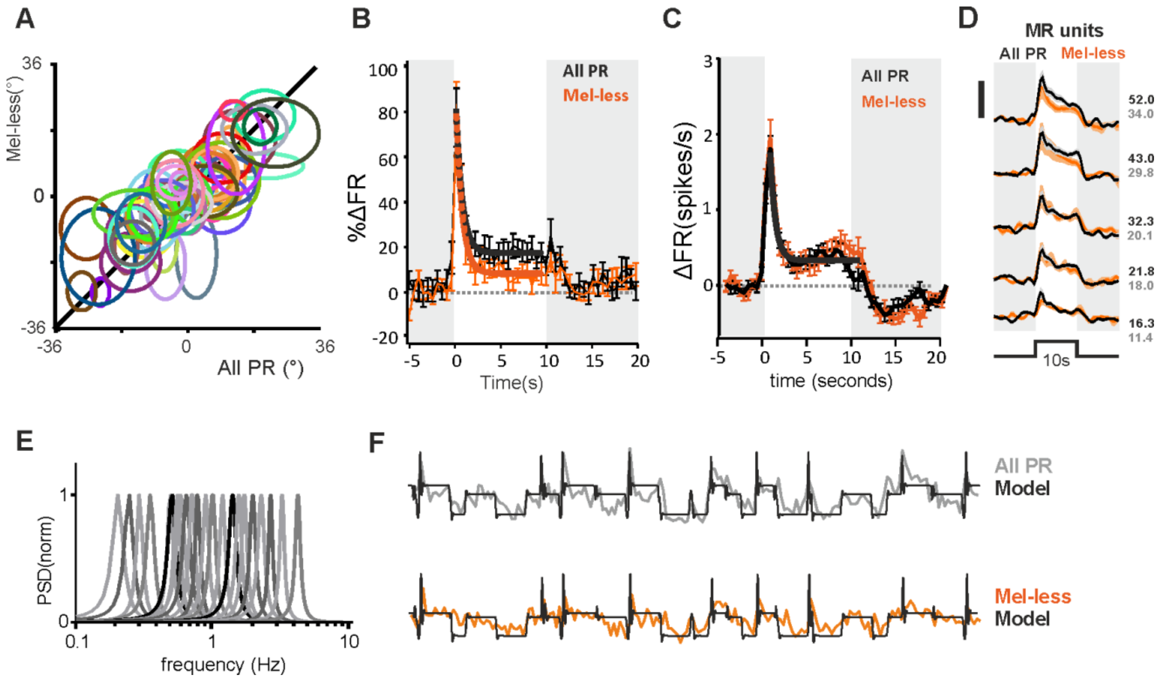

Figure S3. Comparison of responses to 'all-photoreceptor' and 'melanopsin-less' stimuli; Related to Figures 2-4.

A, Ellipses describing location of RFs on azimuth mapped with 'all-photoreceptor' (x-axis) and 'melanopsin-less' (y-axis) stimuli (extent of RF under each condition defined as location on azimuth at half standard deviation on either side of Gaussian fit). Paired two-tailed t-test reveals no significant difference between conditions p=0.59).

B, Mean±SEM change in firing rate as a % of baseline firing rate for MR units (replotted from figure 2), for 'all photoreceptor' (black) and 'melanopsin-less' (orange) bars (10s starting at time 0) presented at RF centre of MR units (n=40). Solid lines show fit for data from 0-10s with exponential decay curves. Separate curves were required for the two conditions (F-test comparison; p<0.0001). Transition between spectra indicated with grey shading.

C, Mean±SEM change in firing rate for 'all-photoreceptor' (black) and 'melanopsin-less' (orange) bars (10s starting at time 0) presented at RF centre of non-MR units (n=40). Solid line shows fit for data from 0-10s with exponential decay curve (data was best fit with a single curve; F-test comparison; p>0.05). Transition between spectra indicated with grey shading.

D, Mean± SEM firing rate (baseline subtracted) of MR units (18 units recorded in 4 *Opn1mw*^{R} mice) at different contrast conditions for 'all-photoreceptor' (black) or 'melanopsin-less' (pink) stimuli. Michelson contrast for melanopsin is shown to right in black, and the mean for rod and cone opsins in grey (%). Scale bar = 5 spikes/s. Transition between spectra indicated with grey shading.

E, PSD of binary modulation stimuli used to model responses across different frequency bands (frequencies spanning 0.2 to 5Hz).

F, Linear filters generated for All-photoreceptor and Melanopsin-less stimuli (Figure 2) were used to predict responses to radiance changes presented during a simulated natural view (Figure 3). Black lines in show predicted responses based on radiance changes for All-photoreceptor and melanopsin-less linear filters, in upper and lower plots, respectively, superimposed upon the mean response of MR units to stimuli rendered in All-photoreceptor or Melanopsin-less spectra (grey and orange lines, respectively). Models predicted actual responses with reasonable accuracy (Pearson's correlation coefficient =0.57 and =0.49, respectively).

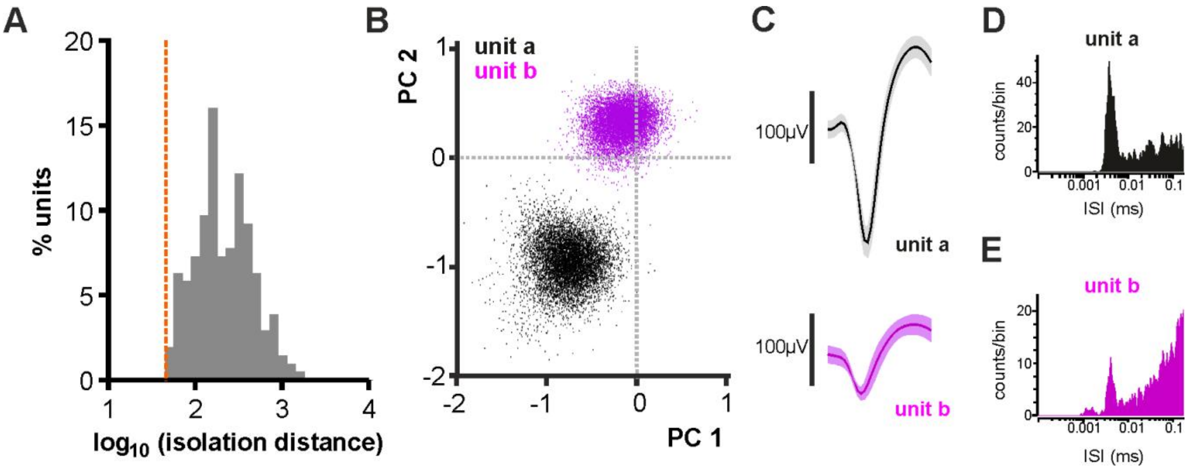

Figure S4. Isolation of single units; Related to STAR Methods.

A, A histogram of isolation distances for all clusters of spikes included in the current dataset. All units exceeded a criterion isolation distance of >50 (indicated with dotted line; used previously as a stringent threshold for unit isolation[1]).

B, Scatter plot shows first two principal components (PC1 and PC2) of representative channel. Black and magenta dots show clusters of spikes assigned as unit 'a' and unit 'b', respectively.

C, Mean±SD of action potential spikes assigned to units a and b in **B** (scale bar = 100µV).

D&E, Log interspike interval (ISI) distributions of spikes assigned to units a and b, respectively.

	LWS opsin effective photons/cm^2/sr/s	SWS opsin effective photons/cm^2/sr/s	Rod opsin effective photons/cm^2/sr/s	Melanopsin effective photons/cm^2/sr/s
Spectrum 1	3.16x10^{13}	3.98x10^{11}	1.00x10^{13}	3.80x10^{12}
Spectrum 2	3.16x10^{13}	3.98x10^{11}	1.00x10^{13}	1.26x10^{13}
Spectrum 3	6.31x10^{13}	6.31x10^{11}	2.00x10^{13}	1.26x10^{13}

Table S1. Effective photon fluxes for spectra 1-3. Related to STAR methods

The effective photon flux for LWS opsin, SWS opsin, Rod opsin and Melanopsin presented by Spectra 1-3.

	LWS opsin Contrast	SWS opsin Contrast	Rod opsin Contrast	Melanopsin Contrast
‘melanopsin-only’ (1 to 2)	<1%	<1%	<1%	52%
‘melanopsin-less’ (2 to 3)	33%	23%	33%	<1%
‘all photoreceptor’ (1 to 3)	33%	23%	33%	52%

Table S2. Estimated contrasts for transitions between each stimulus pair. Related to STAR methods

The Michelson contrast is was calculated for transitions between pairs of spectra; these are summarised for each individual photopigment in the $Opn1mw^R$ retina**.**
